# Expression of hbz mRNA promotes survival in HTLV-1-infected cells with high DNA damage

**DOI:** 10.1186/1742-4690-8-S1-A205

**Published:** 2011-06-06

**Authors:** Karina Flores, Giovanni López, Michael Talledo, Carolina Alvarez, Elsa González, Kristien Verdonck, Eduardo Gotuzzo, Daniel Clark

**Affiliations:** 1 Instituto de Medicina Tropical “Alexander von Humboldt”, Universidad Peruana Cayetano Heredia, Lima, Perú; 2 Facultad de Medicina Alberto Hurtado, Universidad Peruana Cayetano Heredia, Lima, Perú; 3 Laboratorios de Investigación y Desarrollo (LID), Facultad de Ciencias y Filosofía, Universidad Peruana Cayetano Heredia, Lima, Perú

## Background

HTLV1 may exert an initial pro-apoptotic stimulus via Tax-induced DNA damage. On the other hand, Hbz protein antagonizes some Tax effects on gene expression and hbz mRNA has been proposed as essential for maintaining oncogenesis. We hypothesize that expression of the hbz gene contributes to overcoming the pro-apoptotic signals in HTLV1 infected T-cells with DNA damage. To test this hypothesis we studied the expansion of infected cells with high DNA damage and investigated whether hbz mRNA supports survival of those cells.

## Aim and methods

We measured HTLV1 proviral load (PVL) in two groups of HTLV-1-infected subjects showing (1) High DNA damage [HD] (n=18); and (2) Low DNA damage [LD] (n=9). Peripheral blood mononuclear cells were isolated to measure PVL and hbz mRNA levels by real time PCR, and the degree of DNA damage by comet assay (HD: ≥51comets/100cells; LD: ≤50comets/100cells). PVL was expressed as HTLV-1 tax copy number /10^4^ PBMCs. Statistical analyses were based on non-parametric tests.

## Results

PVL was not significantly different between both groups (p=0.959); however, we identified a cluster of high PVL (3168 ±980SD) in the HD group with detectable levels of hbz mRNA. Interestingly, no mRNA expression could be detected in the LD group.

## Conclusion

Our results suggest that hbz mRNA levels rise as DNA damage increases, which is consistent with our hypothesis. The absence of a negative association between PVL and DNA damage along with the detection of hbz mRNA only in HD subjects with high PVL further support our hypothesis.

